# Grating configurations to compress free-electron laser pulses

**DOI:** 10.1107/S160057751701637X

**Published:** 2018-01-01

**Authors:** Luca Poletto, Fabio Frassetto

**Affiliations:** a National Research Council – Institute of Photonics and Nanotechnologies, via Trasea 7, Padova 35136, Italy

**Keywords:** ultrafast technology, free-electron laser, diffraction gratings, chirped-pulse amplification

## Abstract

The optical layout of soft X-ray grating compressors designed to provide both positive and negative group-delay dispersion (GDD) is discussed. They are tailored for chirped-pulse-amplification experiments with seeded free-electron laser sources. Designs with plane or concave gratings are discussed, depending on the sign of the GDD to be introduced.

## Abstract 2

Grating configurations for compressing free-electron-laser pulses are discussed.

## 1. Introduction   

The developments in laser technology over the last decades has led to the generation of pulses as short as a few femtoseconds, providing a unique tool for high-resolution time-domain spectroscopy that has revolutionized many areas of science from solid-state physics to biology (Marciak-Kozlowska & Kozlowski, 2009 ▸). While femtosecond optical lasers have offered unique insights into ultrafast structural dynamics, they are limited by the fact that the structural arrangement and motion of nuclei are not directly accessible from measured optical properties. This scientific gap has been filled by femtosecond sources in the extreme-ultraviolet (XUV) and X-ray regions, such as high-order laser harmonics and free-electron lasers (FELs) (Brabec & Kapteyn, 2008 ▸).

In particular, FEL sources provide spatially coherent radiation in the XUV and X-ray regions with characteristics similar to the light from optical lasers, ultrashort time duration and an increase of six to eight orders of magnitude on the peak brilliance with respect to third-generation synchrotrons. There are several operating FEL facilities already open to users’ experiments: FLASH (http://flash.desy.de), SACLA (http://xfel.riken.jp), LCLS (http://lcls.slac.stanford.edu), FERMI (http://www.elettra.trieste.it), and others are currently close to being opened to users, such as the European XFEL (http://www.xfel.eu) and the SwissFEL (http://www.psi.ch/swissfel).

The handling and conditioning of ultrashort coherent FEL pulses has required the development of suitable optical technologies related to the preservation of the pulse characteristics up to the users’ experiments (Canova & Poletto, 2015 ▸). In particular, this paper is focused on the generation of ultrashort pulses by applying the chirped-pulse-amplification (CPA) technique to FELs.

Several methodologies have been proposed for the generation of pulses on the femtosecond time scale, such as time slicing and reduction of the electron bunch charge (Emma *et al.*, 2004 ▸; Rosenzweig *et al.*, 2008 ▸; Ding *et al.*, 2009 ▸), that have brought about the generation of pulses as short as 3 fs (Ding *et al.*, 2012 ▸). Most of these methods rely on the selection of a small portion of the electron beam which undergoes amplification, that reduces the amount of charge that effectively contributes to the light amplification. This limits the output pulse energy available at the output. More recently, theoretical studies have investigated the possibility to increase the peak power up to the terawatt level (Tanaka, 2015 ▸). However, in all cases, the pulse shortening is constrained by the FEL gain bandwidth. Moreover, none of the proposed methods can control and/or manipulate the spectral and temporal properties of the generated light, which is fundamental in order to achieve fully coherent pulses (Gauthier *et al.*, 2015 ▸).

A different possibility is the optical compression of the pulse generated by the whole electron beam, applying the CPA technique (Strickland & Mourou, 1985 ▸) to seeded FEL pulses (Yu *et al.*, 1994 ▸; Frassetto *et al.*, 2008*a* ▸; Doyuran *et al.*, 2004 ▸; Shu *et al.*, 2011 ▸; Feng *et al.*, 2013 ▸). In this case the electron beam is required to have a non-zero energy chirp in order to generate a chirped pulse, that is later compressed. As for solid-state lasers, where frequency chirping is introduced to stretch the pulse before its amplification and then, after amplification, compensated to recover the short temporal duration and the high peak power, CPA may be applied also to seeded FELs, if the seeding pulse is stretched in time before interacting with the electron beam. This allows the use of the whole electron beam charge obtaining a significantly higher number of photons with ultrashort duration, although requiring a FEL compressor as an additional optical stage.

CPA applied to FEL pulses in the XUV has been experimentally demonstrated for the first time at the FERMI facility (Gauthier *et al.*, 2016 ▸). The experiment was carried out at 37 nm. The FEL has been seeded with a Gaussian laser pulse carrying a linear frequency chirp, that is transmitted to the FEL harmonic pulse generated at the end of the radiator and then compensated by a double-grating compressor. Chirped FEL pulses have been compressed from 140 fs to 50 fs, close to the theoretical Fourier-limited duration of 40 fs. The compressor used at FERMI can be tuned both in group-delay dispersion (GDD) and wavelength, but it gives only negative GDD.

In this paper, different layouts of grating compressors are discussed. Designs with plane or concave gratings are presented, in order to choose the sign of the GDD to be introduced: (1) the configuration with two plane gratings, that gives negative GDD; (2) the configuration with two concave gratings and an intermediate focus between the two, that gives positive GDD; (3) the configuration with one concave grating, one concave mirror and one plane grating, that gives either positive or negative GDD. The techniques to be used to have devices that can be tuned in wavelength and GDD are discussed, in particular the need to have deformable concave gratings.

## 2. Optical configurations for grazing-incidence grating compressor   

### 2.1. Design with two plane gratings and two concave mirrors   

**Figure 1:**
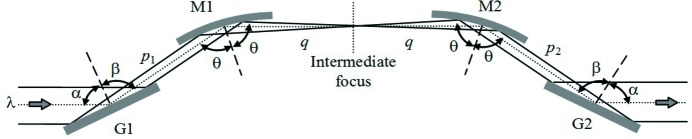
Compressor with two plane gratings and two concave mirrors. Note that the beam comes out parallel to the input only if the subtended angles on the gratings and on the mirrors are the same: α + β = 2θ, that is the particular case shown in the figure.

**Figure 2:**
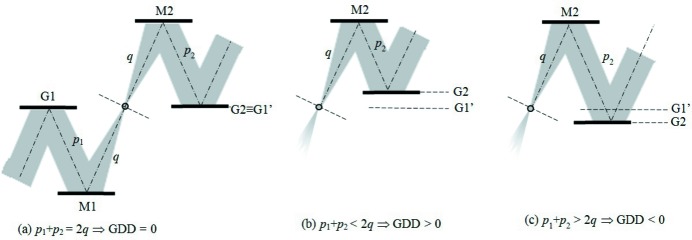
Variation of the GDD in the configuration with two plane gratings and two concave mirrors for different M2–G2 distances: (*a*) zero GDD (G1 is imaged on G2); (*b*) positive GDD (G1 is imaged behind G2); (*c*) negative GDD (G1 is imaged in front of G2).

The problem of introducing a variable GDD has already been studied for the compression of XUV attosecond pulses generated through high-order laser harmonics. The configuration originates from the design of ultrafast devices in the visible and near infrared (Martinez, 1987 ▸) and uses six optical elements, namely four concave mirrors and two plane gratings (Frassetto *et al.*, 2008*b* ▸; Mero *et al.*, 2011 ▸). In any case, a double-grating geometry is required for ultrafast response, since the pulse-front tilt given by the diffraction from a single grating has to be cancelled by a second grating. The configuration is shown in Fig. 1 ▸. It consists of four optical elements: the two gratings, G1 and G2, and the two concave mirrors, M1 and M2, with an intermediate focus. Let *q* indicate the M1-to-focus distance and the focus-to-M2 distance, therefore 2*q* is the total M1–M2 distance. Let *p*_1_ and *p*_2_ indicate the G1–M1 and M2–G2 distances, respectively. Due to the symmetry of the configuration, G1 is imaged on G2 when 2*q* = *p*_1_ + *p*_2_. This is the condition for having the group delay (GD) constant with the wavelength, *i.e.* GDD = 0. For 2*q* > *p*_1_ + *p*_2_, G1 is imaged behind G2 and the resulting GDD is positive. For 2*q* < *p*_1_ + *p*_2_, G1 is imaged in front of G2 and the resulting GDD is negative. The three cases are shown in Fig. 2 ▸.

For a narrow-band pulse with Δλ/λ = Δω/ω < 5%, such as is the case for FELs, the GD is almost linear and given, except for a constant, by
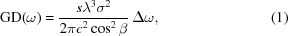
where *s* has to be taken as *s* = 2*q* − *p*_1_ − *p*_2_ with its algebraic sign, λ ≡ 2π*c*/ω is the central wavelength, σ is the grating groove density, β is the diffraction angle from G1 at wavelength λ, *c* is the speed of light in a vacuum and Δω is the frequency extension of the pulse.

The resulting GDD is constant and calculated as
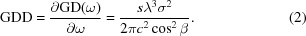
The incidence and diffraction angles on G1 are calculated as
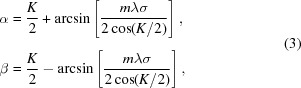
where *K* = α + β is the grating subtended angle and *m* = ±1 is the grating diffraction order. G1 is normally operated in the order *m* = −1, *i.e.* β > α, since this gives a shorter distance *s*, once the GDD has been fixed. For G2, the angles are symmetrical. Both wavelength and GDD are tuned by changing the subtended angle *K*. The sign of the GDD is controlled by changing the distance *p*_2_.

**Figure 3:**
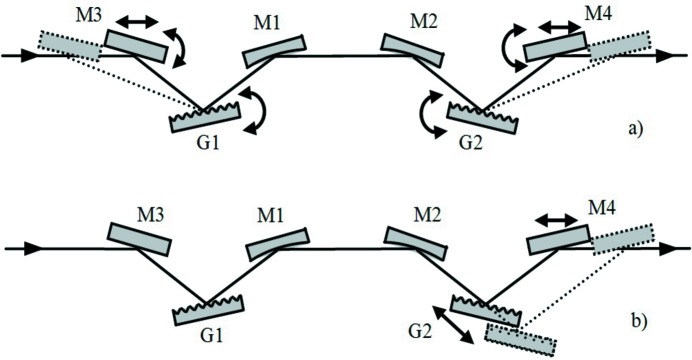
Setup of the compressor with six optical elements. (*a*) The tunability in wavelength and GDD is obtained by changing the angle subtended by the gratings: G1 and G2 are rotated, M3 and M4 are simultaneously rotated and translated. (*b*) The sign of GDD is changed by modifying the M2–G2 distance: G2 and M4 are translated.

The practical setup of the compressor is shown in Fig. 3 ▸. The configuration requires using six optical elements, since two additional plane mirrors have to be inserted before G1 and after G2 for the tunability. The translations and rotations that are needed to change the subtended angle and the sign of the GDD are shown in Figs. 3(*a*) and 3(*b*) ▸, respectively.

In the following paragraphs, three simplified configurations will be discussed: (1) the configuration with two plane gratings, that gives a negative GDD; (2) the configuration with two concave gratings and an intermediate focus between the two, that gives a positive GDD; (3) the configuration with one concave grating, one concave mirror and one plane grating, that gives an either positive or negative GDD.

### 2.2. Design with two plane gratings   

**Figure 4:**
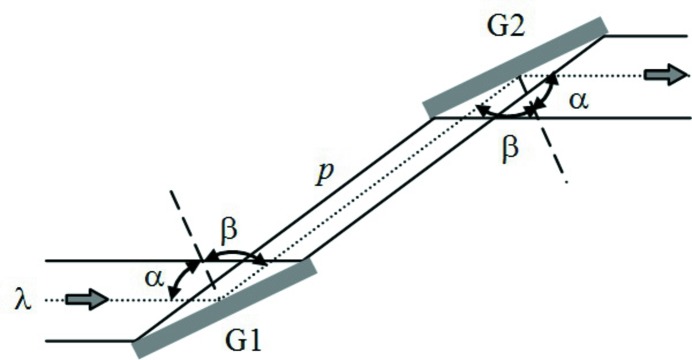
Compressor with two plane gratings for negative GDD. The incidence angle on G2 is equal to the diffraction angle from G1.

The simplest grating compressor adopts two plane gratings at grazing incidence. Here the plane-grating configuration is briefly presented, since it has been already discussed in detail elsewhere (Frassetto & Poletto, 2015 ▸). The setup is shown in Fig. 4 ▸. Two equal plane gratings, G1 and G2, are operated parallel to one another and in opposite diffraction orders, *i.e.* the incidence angle on G2 is equal to the diffraction angle from G1. The second grating G2 compensates both for the pulse-front tilt and for the spectral dispersion introduced by G1, therefore all the rays at different wavelengths exit parallel. The compensating configuration needs to have the two gratings with the diffracting surfaces face to face. It can be shown that the GDD introduced by such a configuration is negative anyway, *i.e.* the optical path increases as the wavelength. For a narrow-band pulse the GDD is calculated by equation (2) where *s* has to be taken as *s* = −*p*.

**Figure 5:**
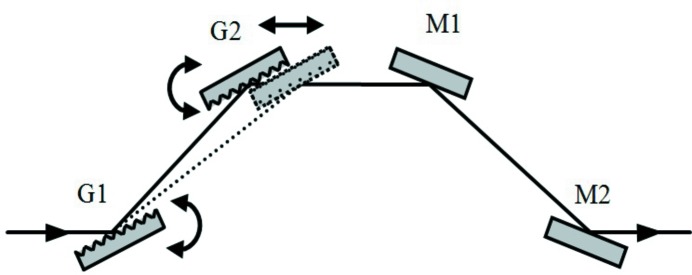
Setup of the compressor with four optical elements: two plane gratings and two plane mirrors. The GDD is negative. The tunability in wavelength and GDD is obtained by changing the angle subtended by the gratings: G1 is rotated, G2 is simultaneously rotated and translated.

The practical setup of the compressor is shown in Fig. 5 ▸. The instrument is tuned in wavelength and GDD by acting on the rotations and translations of the two gratings, in order to change the subtended angles. The two plane mirrors are used to deviate the FEL beam in the same direction as the input.

The front-tilt compensating configuration for two equal gratings requires to have the same illuminated area on the two, therefore the same number of illuminated grooves. The design assumes an input collimated beam. In this case, the compensating configuration is realised when the two gratings are operated at the same subtended angle. The configuration works also when the input beam is diverging, such as in the real case of FELs. In this case, if the gratings are parallel, G2 is illuminated in a length larger than G1, therefore the pulse-front tilt given by the diffraction on G1 is not exactly compensated by G2. The undesired residual pulse-front tilt is corrected by operating G2 at a subtended angle slightly lower than G1, therefore the beam diffracted from G2 is no longer parallel to the input beam. From a practical point of view, the steering of the beam in the original input direction requires to have a translation and rotation on M1 and a rotation on M2, as in the compressor that has been realised for FERMI (Poletto *et al.*, 2015 ▸).

### 2.3. Design with two concave gratings   

**Figure 6:**
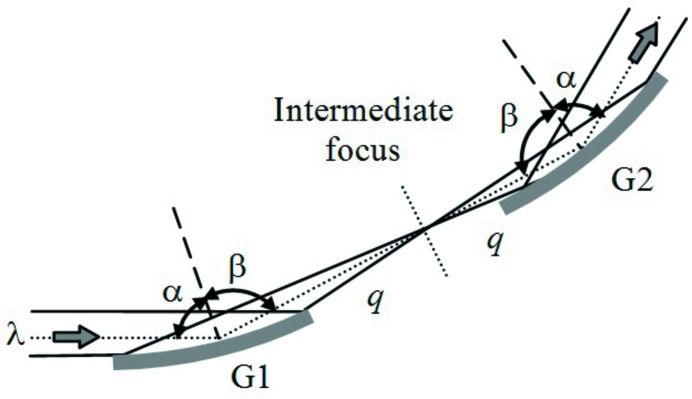
Compressor with two concave gratings for positive GDD. The incidence angle on G2 is equal to the diffraction angle from G1.

With reference to the general configuration shown in Fig. 1 ▸, the functions that are demanded of the concave mirror, *i.e.* the focusing, and of the plane grating, *i.e.* the diffraction, can be realised on a single optical element, namely a concave grating. The configuration is shown in Fig. 6 ▸. The gratings are used in the compensating geometry and have the same radius to give a symmetric configuration with an intermediate focus that is half way between the two. The compensating configuration needs to have the two gratings with the diffracting surfaces facing in the same direction.

Let *q* indicate the G1-to-focus and the focus-to-G2 distances, that have to be the same for the correction of the pulse-front tilt. The GDD is calculated from equation (2), where the value *s* to be used in the formula is *s* = 2*q*. The GDD is positive, *i.e.* the optical path is decreasing when the wavelength is increasing.

The grating radius *R* is calculated from the equation for the spectral focusing at wavelength λ,
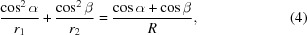
where *r*_1_ and *r*_2_ are, respectively, the grating entrance and exit arms.

For a parallel beam (*r*_1_ = ∞) and *r*_2_ = *q*, it results that *R* = *q*(cosα + cosβ)/cos^2^β for both gratings. For a diverging beam, it is preferable that the compressor does not alter the beam divergence at its output, *i.e.* the beam exiting G2 has to have the same divergence as the beam at the input. This is realised if G2 makes a virtual focus in the position of the FEL source. The radii of the two gratings *R*_1_ and *R*_2_ are calculated as
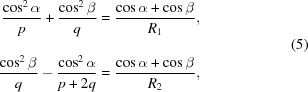
where *p* is the distance from the FEL source to G1.

**Figure 7:**
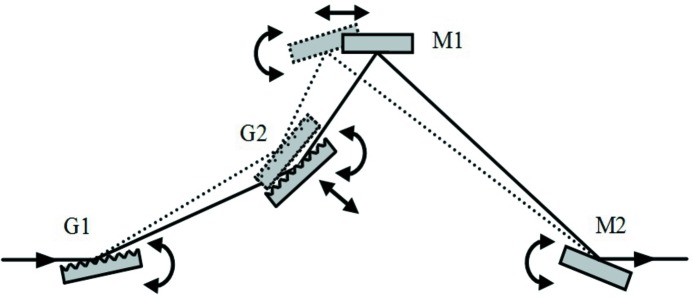
Setup of the compressor with four optical elements: two concave gratings and two plane mirrors. The GDD is positive. The tunability in wavelength and GDD is obtained by changing the angle subtended by the two gratings: G1 and M2 are rotated, G2 and M1 are simultaneously rotated and translated.

The practical setup of the compressor is shown in Fig. 7 ▸. The operation at different subtended angles requires both G1 and G2 to be rotated and simultaneously G2 to be translated. The two plane mirrors M1 and M2 are used to deviate the FEL beam in the same direction as the input.

When the subtended angle is changed, the grating radii have to be varied according to equations (5) to keep the spectral focusing in a fixed position. This can be done through active diffraction gratings where the radius is changed by deforming the grating substrate. The realisation of active gratings has been extensively reported in the literature for a wide set of applications, ranging from telecommunication and optical displays in the visible (Wong *et al.*, 2003 ▸; Guerrero *et al.*, 2010 ▸) to synchrotron beamlines in the soft X-ray region (Chen *et al.*, 2001 ▸; Fung *et al.*, 2007 ▸). In particular, it is mentioned here that active gratings have already been realised for use in grazing-incidence monochromators for the spectral selection of ultrashort pulses (Frassetto *et al.*, 2013 ▸). The grating consists of a silicon deformable thin substrate, on the top of which a periodic laminar profile is realised by UV lithography. The passive substrate is glued to a piezoelectric (PZT) disc. The PZT expansion against the passive substrate generates an almost cylindrical change in the surface shape, with radius controlled by the voltage applied to its electrodes (Bonora *et al.*, 2012 ▸). A similar device could be used to realise compressors with active concave gratings.

Similarly to the compressor with plane gratings, where the GDD to be introduced is only negative, the compressor with concave gratings gives only positive GDD. Both configurations have the minimum number of optical elements, namely two gratings and two mirrors.

### 2.4. Design with one concave grating, one concave mirror and one plane grating   

**Figure 8:**
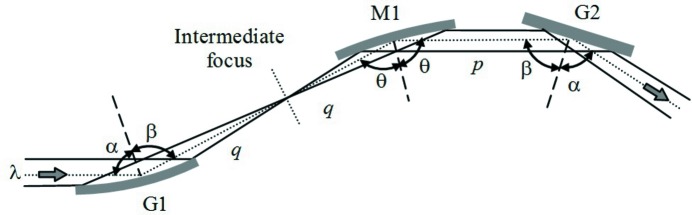
Compressor with one concave grating, one concave mirror and one plane grating. Note that the beam exits M1 parallel to the input only if the subtended angles on the G1 and M1 are the same: α + β = 2θ, that is the particular case shown in the figure.

The double-grating configurations discussed above give a variable GDD but with fixed sign, either negative (plane gratings) or positive (concave gratings). A three-element configuration is discussed here, where the sign of the GDD can be arbitrarily chosen. The layout is shown in Fig. 8 ▸. It is a simplified version of the general configuration shown in Fig. 1 ▸. The light is diffracted and focused by the concave grating G1 on the intermediate focus, then it is collimated by the concave mirror M1 and finally dispersed by the plane grating G2. Let *q* indicate the G1-to-focus and the focus-to-M1 distances and *p* the M1–G2 distance. The GDD is calculated from equation (2), where the value *s* to be used is *s* = 2*q* − *p*. The sign of the GDD can be controlled by translating G2 with respect to M1: GDD < 0 for 2*q* < *p*, GDD > 0 for 2*q* > *p*. When the subtended angle is changed, the radius of G1 has to be varied according to equation (5).

**Figure 9:**
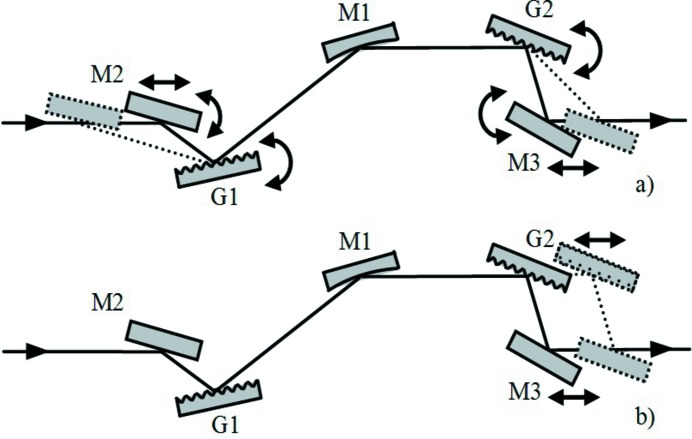
Setup of the compressor with five optical elements. (*a*) The tunability in wavelength and GDD is obtained by changing the angle subtended by the gratings: G1 and G2 are rotated, M2 and M3 are simultaneously rotated and translated. (*b*) The sign of GDD is changed by modifying the M1–G2 distance: G2 and M3 are translated.

The practical setup of the compressor is shown in Fig. 9 ▸. The configuration has five optical elements, since two additional plane mirrors have to be inserted before G1 and after G2 for the tunability. The translations and rotations that are needed to change the subtended angle and the sign of the GDD are shown in Figs. 9(*a*) and 9(*b*) ▸, respectively.

## 3. Spatial chirp   

All the configurations presented above give a spatial chirp of the output pulse, *i.e.* rays with different wavelengths have the same output direction but they are not overlapped. In the conventional design of grating compressors for optical pulses, the spatial chirp is cancelled by making the beam pass twice though the same gratings, so the output spatial dispersion is zero. This cannot be realised in grazing incidence, since it would require the insertion of two additional gratings that will reduce the efficiency to unacceptable low values.

The spatial chirp may degrade the quality of the final focus after the compressor in the case of a divergent beam, since different wavelengths are focused in different points in the direction of the spectral dispersion. This broadens the spot size in one direction, giving a slight asymmetry in the spot profile. However, it has been shown by simulations (Frassetto & Poletto, 2015 ▸) and in the real experiment (Gauthier *et al.*, 2016 ▸) that the degradation of the spot size is typically below 10 µm, therefore it can normally be neglected.

## 4. Design of a compressor for 10 nm wavelength   

As a test case, we discuss the design of a grating compressor for a spectral interval centred at 10 nm using two plane or two concave gratings. The groove density is 1.200 grooves mm^−1^, the G1–G2 distance is 800 mm and the subtended angle is selectable in the 158°–168° interval. The parameters of the FEL source are assumed to be similar to those of FERMI: ∼200 µm r.m.s. source size and 12.5 µrad r.m.s. divergence at 10 nm. The FEL bandwidth is that of a Fourier-transform-limited pulse of 10 fs, therefore the chirp to be compensated by the compressor is presumably below 120–150 fs. The compressor is assumed to be 50 m from the source, which is a realistic value for FEL beamlines.

**Figure 10:**
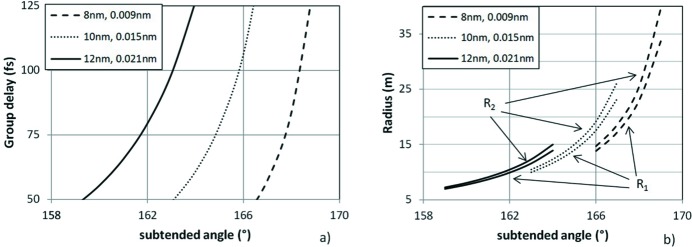
Parameters of the grating compressor for an 8–12 nm interval. (*a*) Group delay as a function of the subtended angle for three wavelengths. The pulse bandwidth is that of a 10 fs transform-limited pulse. (*b*) Radii *R*_1_ and *R*_2_ of concave gratings.

The GD delay within the pulse bandwidth is shown in Fig. 10(*a*) ▸ at different subtended angles and wavelengths. Once the subtended angle has been chosen, the GDD given by the compressor in the two configurations (plane or concave gratings) is the same, but with opposite signs. The radii of the concave gratings are calculated from equation (5) and are shown in Fig. 10(*b*) ▸. The gratings have to be deformed from several meters to a few tens of meters, depending on the wavelength and on the delay to be compensated.

Both configurations have been simulated by ray-tracing. In particular, the results at 10 nm wavelength for GD ≃ 100 fs (GDD ≃ ±190 fs^2^) to be introduced by the compressor are resumed in Table 1 ▸. The results obtained at different wavelength and GDDs are similar in terms of optical performance and are not presented here. The double-grating configuration is very effective in compensating for the pulse-front tilt due to the diffraction from G1, from almost 900 fs to a few femtoseconds. The residual distortion of the pulse-front due to the optical configuration is almost negligible for plane gratings (<1 fs) and slightly higher for concave gratings due the residual coma aberration given by the concave surfaces, but in any case below 3 fs. In practice, the residual distortion is limited by the surface quality of the optical surfaces and has to be calculated once the real surface parameters are given. In the case discussed here, tangential slope errors lower than 3 µrad r.m.s., well within the present capabilities of optical manufacturing, are required to have a residual front-tilt distortion below 10 fs. Note that the two plane gratings are operated at slightly different subtended angles, due to the need to compensate for the beam divergence.

The focal point of the beamline has been assumed to be a Kirkpatrick–Baez focusing system that is placed 40 m from the compressor with a focal length of 1.5 m. Under these conditions the resulting spatial chirp within the pulse bandwidth in the focus is lower than 1 µm, and is therefore totally negligible.

## 5. Conclusions   

The design of grating compressors to be applied to XUV ultrashort chirped pulses has been presented. Different configurations have been discussed, starting from the general layout that requires two plane gratings and two concave mirrors, similarly to what is normally realised in grating compressors for optical pulses. In particular, the simplest design adopts two gratings that may be plane or concave, depending on the sign of the GDD to be introduced.

All the configurations discussed here are based on the use of grazing-incidence reflective gratings in the classical diffraction geometry. The use of ruled gratings restricts the photon energies for which this approach is useful, since the grating efficiency decreases dramatically for wavelengths shorter than ∼4 nm. However, it can be applied to chirped pulse amplification of seeded FELs operated in the XUV and soft X-rays.

## Figures and Tables

**Table 1:** Parameters of the ray-tracing simulations of the grating compressor

General	
Wavelength of operation	10 nm
Bandwidth	0.0147 nm
Source-to-G1 distance	50.000 mm
G1–G2 distance	800 mm
Grating groove density	1.200 grooves mm^−1^
Group delay within the bandwidth	100 fs
Group delay dispersion	±190 fs^2^

Plane grating configuration	
Subtended angle on G1	166.0°
Subtended angle on G2	165.5°
Pulse-front tilt after diffraction from G1	890 fs
Residual pulse-front distortion at the output	<1 fs

Concave grating configuration	
Subtended angle on G1 and G2	166.0°
Radius of G1	17.57 m
Radius of G2	19.17 m
Pulse-front tilt after diffraction from G1	890 fs
Residual pulse-front distortion at the output	<3 fs

## References

[bb1] Bonora, S., Frassetto, F., Zanchetta, E., Della Giustina, G., Brusatin, G. & Poletto, L. (2012). *Rev. Sci. Instrum.* **83**, 123106. 10.1063/1.4770333 23277971

[bb2] Brabec, T. & Kapteyn, H. (2008). *Strong Field Laser Physics.* Berlin: Springer.

[bb3] Canova, F. & Poletto, L. (2015). *Optical Technologies for Extreme-Ultraviolet and Soft X-ray Coherent Sources.* New York: Springer.

[bb4] Chen, S.-J., Chen, C. T., Perng, S. Y., Kuan, C. K., Tseng, T. C. & Wang, D. J. (2001). *Nucl. Instrum. Methods Phys. Res. A*, **467–468**, 298–301.

[bb6] Ding, Y., Decker, F. J., Emma, P., Feng, C., Field, C., Frisch, J., Huang, Z., Krzywinski, J., Loos, H., Welch, J., Wu, J. & Zhou, F. (2012). *Phys. Rev. Lett.* **109**, 254802. 10.1103/PhysRevLett.109.254802 23368472

[bb5] Ding, Y., Huang, Z., Ratner, D., Bucksbaum, P. & Merdji, H. (2009). *Phys. Rev. ST Accel. Beams*, **12**, 060703.

[bb7] Doyuran, A., DiMauro, L., Graves, W., Heese, R., Johnson, E. D., Krinsky, S., Loos, H., Murphy, J. B., Rakowsky, G., Rose, J., Shaftan, T., Sheehy, B., Shen, Y., Skaritka, J., Wang, X., Wu, Z. & Yu, L. H. (2004). *Nucl. Instrum. Methods Phys. Res. A*, **528**, 467–470. 10.1103/PhysRevLett.91.074801 12935021

[bb8] Emma, P. J., Bane, K., Cornacchia, M., Huang, Z., Schlarb, H., Stupakov, G. & Walz, D. (2004). *Phys. Rev. Lett.* **92**, 074801. 10.1103/PhysRevLett.92.074801 14995861

[bb9] Feng, C., Shen, L., Zhang, M., Wang, D., Zhao, Z. & Xiang, D. (2013). *Nucl. Instrum. Methods Phys. Res. A*, **712**, 113–119.

[bb10] Frassetto, F., Bonora, S., Vozzi, C., Stagira, S., Zanchetta, E., Della Giustina, G., Brusatin, G. & Poletto, L. (2013). *Opt. Express*, **21**, 12996–13004. 10.1364/OE.21.012996 23736553

[bb11] Frassetto, F., Giannessi, L. & Poletto, L. (2008*a*). *Nucl. Instrum. Methods Phys. Res. A*, **593**, 14–16.

[bb12] Frassetto, F. & Poletto, L. (2015). *Appl. Opt.* **54**, 7985–7992. 10.1364/AO.54.007985 26368974

[bb13] Frassetto, F., Villoresi, P. & Poletto, L. (2008*b*). *Opt. Express*, **16**, 6652–6667. 10.1364/oe.16.006652 18545369

[bb14] Fung, H. S., Yuh, J. Y., Huang, L. J., Tseng, T. C., Perng, S. Y., Wang, D. J., Tsang, K. L. & Chung, S. C. (2007). *AIP Conf. Proc.* **879**, 563–566.

[bb16] Gauthier, D., Allaria, E., Coreno, M., Cudin, I., Dacasa, H., Danailov, M. B., Demidovich, A., Di Mitri, S., Diviacco, B., Ferrari, E., Finetti, P., Frassetto, F., Garzella, D., Künzel, S., Leroux, V., Mahieu, B., Mahne, N., Meyer, M., Mazza, T., Miotti, P., Penco, G., Raimondi, L., Ribič, P. R., Richter, R., Roussel, E., Schulz, S., Sturari, L., Svetina, C., Trovò, M., Walker, P. A., Zangrando, M., Callegari, C., Fajardo, M., Poletto, L., Zeitoun, P., Giannessi, L. & De Ninno, G. (2016). *Nat. Commun.* **7**, 13688. 10.1038/ncomms13688 PMC5146278 27905401

[bb15] Gauthier, D., Ribič, P. R., De Ninno, G., Allaria, E., Cinquegrana, P., Danailov, M. B., Demidovich, A., Ferrari, E., Giannessi, L., Mahieu, B. & Penco, G. (2015). *Phys. Rev. Lett.* **115**, 114801. 10.1103/PhysRevLett.115.114801 26406834

[bb17] Guerrero, R. A., Sze, M. W. C. & Batiller, J. R. A. (2010). *Appl. Opt.* **49**, 3634–3639. 10.1364/AO.49.003634 20648128

[bb18] Marciak-Kozlowska, J. & Kozlowski, M. (2009). *From Femto-to Attoscience and Beyond.* Hauppauge: Nova Science Publishers.

[bb19] Martinez, O. (1987). *IEEE J. Quantum Electron.* **23**, 1385–1387.

[bb20] Mero, M., Frassetto, F., Villoresi, P., Poletto, L. & Varjú, K. (2011). *Opt. Express*, **19**, 23420–23428. 10.1364/OE.19.023420 22109218

[bb21] Poletto, L., Frassetto, F., Miotti, P., Gauthier, D., Fajardo, M., Mahieu, B., Svetina, C., Zangrando, M., Zeitoun, P. & De Ninno, G. (2015). *Proc. SPIE*, **9512**, 951210.

[bb23] Rosenzweig, J. B., Alesini, D., Andonian, G., Boscolo, M., Dunning, M., Faillace, L., Ferrario, M., Fukusawa, A., Giannessi, L., Hemsing, E., Marcus, G., Marinelli, A., Musumeci, P., O’Shea, B., Palumbo, L., Pellegrini, C., Petrillo, V., Reiche, S., Ronsivalle, C., Spataro, B. & Vaccarezza, C. (2008). *Nucl. Instrum. Methods Phys. Res. A*, **593**, 39–44.

[bb24] Shu, X., Peng, T. & Dou, Y. (2011). *J. Electron Spectrosc. Relat. Phenom.* **184**, 350–353.

[bb25] Strickland, D. & Mourou, G. (1985). *Opt. Commun.* **56**, 219–221.

[bb26] Tanaka, T. (2015). *Phys. Rev. Lett.* **110**, 084801.

[bb27] Wong, C. W., Jeon, Y., Barbastathis, G. & Kim, S. G. (2003). *Appl. Opt.* **42**, 621–626. 10.1364/ao.42.000621 12564480

[bb28] Yu, L. H., Johnson, E., Li, D. & Umstadter, D. (1994). *Phys. Rev. E*, **49**, 4480–4486. 10.1103/physreve.49.4480 9961743

